# The role of systemic immune-inflammation index in predicting pathological complete response of breast cancer after neoadjuvant therapy and the establishment of related predictive model

**DOI:** 10.3389/fonc.2024.1437140

**Published:** 2024-11-01

**Authors:** Ziyue Zhang, Yixuan Zeng, Wenbo Liu

**Affiliations:** ^1^ Faculty of Medicine, Debrecen University, Debrecen, Hungary; ^2^ Faculty of Medicine, University of Bonn, Bonn, Germany; ^3^ Department of Plastic and Cosmetic Maxillofacial Surgery, The First Affiliated Hospital of Xi’an Jiaotong University, Xi’an, China

**Keywords:** breast cancer, neoadjuvant chemotherapy, pathological complete response, nomogram, prediction model

## Abstract

**Objective:**

To investigate the role of systemic immune-inflammation index (SII) in complete pathological response (pCR) of breast cancer patients after neoadjuvant chemotherapy, and to establish and validate a nomogram for predicting pCR.

**Methods:**

Breast cancer patients were selected from the First Affiliated Hospital of Xi’an Jiaotong University from January 2020 to December 2023. The optimal cut-off value of SII was calculated via ROC curve. The correlation between SII and clinicopathological characteristics was analyzed by Chi-square test. Logistic regression analysis was performed to evaluate the factors that might affect pCR. Based on the results of Logistic regression analysis, a nomogram for predicting pCR was established and validated.

**Results:**

A total of 112 breast cancer patients were included in this study. 33.04% of the patients achieved pCR after neoadjuvant therapy. Chi-square test showed that SII was significantly correlated with pCR (P=0.001). Logistic regression analysis suggested that Ki-67 (P=0.039), therapy cycle (P<0.001), CEA (P=0.025) and SII (P=0.019) were independent predictors of pCR after neoadjuvant chemotherapy. A nomogram based on Ki-67, therapy cycle, CEA and SII showed a good predictive ability.

**Conclusion:**

Ki-67, therapy cycle, CEA and SII were independent predictors of pCR of breast cancer after neoadjuvant chemotherapy. The nomogram based on the above positive factors showed a good predictive ability.

## 1 Introduction

Breast cancer is the most common malignant tumor in the world, with an estimated 2.3 million new cases of breast cancer in 2020, accounting for 11.7% of all new cancers (1). In the past few decades, researchers have conducted a series of clinical studies to establish a standard treatment for breast cancer (2–4). Studies have shown that neoadjuvant therapy can significantly prolong the overall survival of breast cancer patients, which makes neoadjuvant chemotherapy a recommended treatment for advanced breast cancer (5, 6). Preoperative or neoadjuvant therapy, including targeted therapy or immunotherapy, has become the standard treatment for most early HER-2^-^ and triple-negative breast cancer. Studies have shown that pathological complete response (pCR) after neoadjuvant therapy is significantly correlated with the prognosis of patients (7, 8). Therefore, it is necessary to clarify the factors that may affect pCR after neoadjuvant immunotherapy and establish relevant models for predicting therapy efficacy.

Previous studies have shown that the immune system plays an important role in the treatment response and prognosis of breast cancer (9, 10). As a key part of the host immune system, peripheral blood inflammation indicators, including neutrophil-to-lymphocyte ratio (NLR), platelet-to-lymphocyte ratio (PLR), lymphocyte-to-monocyte ratio (LMR) and systemic immune inflammation index (SII), are considered to be significantly associated with poor prognosis of malignant tumors (11–13). Moreover, these indicators can reflect the efficacy and prognosis of breast cancer patients receiving neoadjuvant therapy (12, 14–17). Among them, SII can well reflect the body’s immune response and inflammatory status. Although SII has been confirmed to be related to the therapeutic effect and prognosis of patients with other malignant tumors (18–20), its correlation with the therapeutic effect and prognosis of breast cancer patients remains to be further studied.

Therefore, the purpose of this study was to clarify the predictive effect of SII on pCR after neoadjuvant chemotherapy for breast cancer. At the same time, a nomogram prediction model based on SII for the pCR of neoadjuvant immunotherapy for breast cancer was established and verified.

## 2 Methods

### 2.1 Participants

A total of 112 breast cancer patients who were treated in the First Affiliated Hospital of Xi’an Jiaotong University from January 2020 to December 2023 were included in this study according to the inclusion criteria and exclusion criteria. Inclusion criteria are as follows: 1) preoperative pathological diagnosis of breast cancer; 2) radical surgery after neoadjuvant chemotherapy; 3) the clinicopathological data and postoperative pathological data of the patients were complete. The exclusion criteria are as follows: 1) received other anti-tumor treatment before neoadjuvant chemotherapy; 2) withdrawal from neoadjuvant therapy; 3) refused surgical treatment; 4) the clinicopathological data were incomplete. This study adhered to the Declaration of Helsinki, and was approved and supervised by the Ethics Committee of the First Affiliated Hospital of Xi’an Jiaotong University (No. XJTU1AF2022LSK-335). Because this research is a retrospective study, the Ethics Committee of the First Affiliated Hospital of Xi’an Jiaotong University waived the need for informed consent from the patients.

### 2.2 Data collection and processing

Baseline data, clinicopathological data, treatment-related data and pre-treatment laboratory examination data were collected. Data processing was performed using Microsoft Excel and SPSS26.0 software. The optimal cut-off values of menarche age, primiparous age, Ki-67, therapy cycle, NLR, PLR, LMR and SII (SII = platelet count × lymphocyte count/white blood cell count) were calculated according to the receiver operating characteristic curve (ROC). Then, the above continuous variables are converted into binary variables according to the optimal cut-off value. The remaining hematological parameters (including CEA, CA153, HGB, WBC, platelet, lymphocyte, neutrophil, monocyte and albumin) were converted into categorical variables according to the normal range values.

### 2.3 Statistical analysis

SPSS26.0 and RStudio software were used for statistical analysis. The difference between the two groups was tested by Chi-square test. Univariable and multivariable Logistic regression analyses were used to identify factors that might be related to pCR after neoadjuvant therapy. According to the results of multivariable Logistic regression analysis, a nomogram for predicting pCR after neoadjuvant chemotherapy of breast cancer was established. ROC curve, Bootstrap calibration curve, Decision curve analysis (DCA) and Clinical impact curve (CIC) were used to verify the predictive ability of the constructed nomogram. P<0.05 was considered statistically significant.

## 3 Results

### 3.1 Baseline characteristics of enrolled patients

A total of 112 breast cancer patients were included in this study, with a median age of 53 (range: 34-72) years. All patients received preoperative neoadjuvant chemotherapy and radical surgery. Postoperative pathology confirmed that 33.04% (37/112) of patients achieved pCR. The optimal cut-off value of SII calculated by ROC curve was 598.5. According to the optimal cut-off value, we divided the patients into high SII group (SII≥598.5) and low SII group (SII<589.5), and analyzed the relationship between SII and the basic clinicopathological data of the patients. The results showed that there was no significant correlation between SII and the general characteristics of patients, but it was significantly correlated with the pCR rate of patients (X^2^ = 11.11, P=0.001) (**Table 1**).

**Table 1:** Baseline features.

Items		SII<598.5	SII≥598.5	Total	X^2^	P
**Age (Years)**	18-44	7	11	18	1.91	0.423
	45-59	25	44	69		
	60-74	13	12	25		
**cT**	1	5	12	17		0.358^a^
	2	33	42	75		
	3	1	6	7		
	4	6	7	13		
**cN**	1	9	6	15		0.230^a^
	2	23	38	61		
	2	7	27	34		
	3	6	6	12		
**cM**	0	43	59	102		0.311^a^
	x	2	8	10		
**Clinical stage (AJCC)**	II	31	38	69	1.69	0.236
	III	14	29	43		
**Pathological type**	IDC	43	65	108		1.000^a^
	Other	2	2	4		
**Grade**	2	33	52	85	0.27	0.656
	3	12	15	27		
**ER**	–	21	24	45	1.32	0.326
	+	24	43	67		
**PR**	–	21	27	48	0.45	0.562
	+	24	40	64		
**HER-2**	–	7	14	21	1.21	0.752
	1+	16	18	34		
	2+	7	10	17		
	3+	15	25	40		
**Ki-67**	<43	22	25	47	1.48	0.246
	≥43	23	42	65		
**Molecular classification**	HER-2^+^	18	19	37		0.574^a^
	Luminal A	2	1	3		
	Luminal B (HER-2^-^)	4	8	12		
	Luminal B (HER-2^+^)	19	35	54		
	Triple negative	2	5	7		
**Regimen**	CEF	1	3	4		0.701^a^
	CET-T	2	0	2		
	DCH-TH	2	7	9		
	DC-T	1	4	5		
	EC-5	1	1	2		
	EC-TH	23	29	52		
	EC-T	6	11	17		
	TCH	4	3	7		
	TEC	3	6	9		
	TH	2	3	5		
**Therapy cycle**	≥6	19	37	56	1.82	0.247
	<6	26	30	56		
**pCR**	No	22	53	75	11.11	**0.001**
	Yes	23	14	37		

a Fisher exact probability method. The bold values mean “P<0.05”. IDC, Invasive ductal carcinoma; ER, Estrogen receptor; PR, Progesterone receptor; HER-2, Human epidermal growth factor receptor-2; pCR, Pathological complete response.

### 3.2 Analysis of the influencing factors of pCR after neoadjuvant chemotherapy for breast cancer

In order to clarify the factors that might affect the pCR after neoadjuvant therapy for breast cancer, we performed a univariable Logistic regression analysis of the patient’s clinicopathological data and hematological parameters. The results showed that menarche age (P=0.004), PR (P=0.013), Ki-67 (P<0.001), molecular classification [Luminal B (HER-2^-^), P=0.041], therapy cycle (P<0.001), CEA (P=0.001), CA153 (P<0.001), PLR (P=0.013), LMR (P=0.014) and SII (P =0.001) were significantly correlated with pCR after neoadjuvant therapy (**Table 2**). The above positive indicators were further analyzed by multivariable regression analysis. It was showed that Ki-67 (P=0.039), therapy cycle (P<0.001), CEA (P=0.025) and SII (P=0.019) were independent predictors of pCR after neoadjuvant chemotherapy in breast cancer patients (**Table 3**).

**Table 2:** Univariable Logistic regression analysis.

Factors		Univariable analysis			
		OR	95% CI		P
			Lower	Upper	
**Age (Years)**	18-44	1.00			
	45-59	1.14	0.36	3.60	0.826
	60-74	2.04	0.56	7.49	0.281
**Menarche age (Years)**	<13	1.00			
	≥13	3.49	1.49	8.17	**0.004**
**Menstrual states**	No	1.00			
	Yes	1.67	0.64	4.37	0.299
**Primiparous age (Years)**	<23	1.00			
	≥23	1.09	0.49	2.39	0.838
**cT**	1	1.00			
	2	1.94	0.57	6.52	0.286
	3	4.33	0.67	28.11	0.124
	4	0.27	0.03	2.78	0.271
**cN**	0	1.00			
	1	1.21	0.37	3.99	0.753
	2	0.40	0.09	1.83	0.237
	3	1.43	0.30	6.88	0.656
**cM**	0	1.00			
	x	0.48	0.10	2.38	0.367
**Clinical stage (AJCC)**	II	1.00			
	III	0.57	0.25	1.32	0.188
**Pathological type**	IDC	1.00			
	Other	1.50	0.15	14.94	0.730
**Grade**	2	1.00			
	3	0.00	0.00		0.998
**ER**	–	1.00			
	+	0.36	0.16	0.81	**0.013**
**PR**	–	1.00			
	+	0.70	0.32	1.56	0.385
**HER-2**	–	1.00			
	1+	1.53	0.45	5.26	0.499
	2+	1.33	0.31	5.67	0.697
	3+	2.13	0.65	6.99	0.211
**Ki-67 (%)**	<43	1.00			
	≥43	0.20	0.09	0.47	**0.000**
**Molecular classification**	HER-2^+^	1.00			
	Luminal A	2.35	0.20	28.27	0.50
	Luminal B (HER-2^-^)	0.11	0.01	0.92	**0.04**
	Luminal B (HER-2^+^)	0.42	0.17	1.03	0.06
	Triple negative	0.88	0.17	4.51	0.88
**Regimen**	CEF	1.00			
	CET-T	3.00	0.08	107.45	0.547
	DCH-TH	3.75	0.27	51.37	0.322
	DC-T	0.75	0.03	17.51	0.858
	EC-5	3.00	0.08	107.45	0.547
	EC-TH	1.33	0.13	13.82	0.809
	EC-T	1.64	0.14	19.39	0.696
	TCH	0.50	0.02	11.09	0.661
	TEC	2.40	0.18	32.88	0.512
	TH	0.75	0.03	17.51	0.858
**Therapy cycle**	≥6	1.00			
	<6	99.00	12.72	770.45	**0.000**
**CEA (ng/mL)**	≤5	1.00			
	>5	0.03	0.00	0.26	**0.001**
**CA153 (U/mL)**	≤25	1.00			
	>25	0.14	0.05	0.39	**0.000**
**HGB (g/L)**	<110	1.00			
	110-150	2.06	0.22	19.09	0.526
	>150	0.00	0.00		1.000
**WBC (×10^9^/L)**	<3.5	1.00			
	3.5-9.5	0.00	0.00		0.999
	>9.5	0.00	0.00		0.999
**Platelet (×10^9^/L)**	<100	1.00			
	100-300	877984479.78	0.00		0.999
	>300	717996196.80	0.00		0.999
**Lymphocyte (×10^9^/L)**	<0.8	1.00			
	0.8-4	1.50	0.15	14.94	0.730
**Neutrophil (×10^9^/L)**	<1.5	1.00			
	1.5-8	819435978.78	0.00		1.000
	>8	646183800.41	0.00		1.000
**Monocyte (×10^9^/L)**	<0.12	1.00			
	0.12-0.8	0.49	0.03	8.00	0.614
**Albumin (g/L)**	<30	1.00			
	30-50	866267971.84	0.00		0.999
**NLR**	<2.02	1.00			
	≥2.02	0.34	0.15	0.80	**0.013**
**PLR**	>162	1.00			
	≥162	0.35	0.15	0.81	**0.014**
**LMR**	<6.4	1.00			
	≥6.4	1.38	0.60	3.14	0.449
**SII**	<598.5	1.00			
	≥598.5	0.25	0.11	0.58	**0.001**

The bold values mean “P<0.05”. OR, Odds ratio; CI, Confidence interval; IDC, Invasive ductal carcinoma; ER, Estrogen receptor; PR, Progesterone receptor; HER-2, Human epidermal growth factor receptor-2; CEA, Carcinoembryonic antigen; CA153, Carbohydrate antigen 153; HGB, Hemoglobin; WBC, White blood cell; NLR, Neutrophil-to-lymphocyte ratio; PLR, Platelet-to-lymphocyte ratio; LMR, Lymphocyte-to-monocyte ratio; SII, Systemic immune inflammation index.

**Table 3:** Multivariable Logistic regression analysis.

Factors		Multivariable analysis			
		OR	95%CI		P
			Lower	Upper	
**Ki-67 (%)**	<43	1.00			
	≥43	0.26	0.07	0.94	**0.039**
**Therapy cycle**	≥6	1.00			
	<6	94.95	10.04	897.67	**0.000**
**CEA (ng/mL)**	≤5	1.00			
	>5	0.08	0.01	0.73	**0.025**
**SII**	<598.5	1.00			
	≥598.5	0.19	0.05	0.76	**0.019**

The bold values mean “P<0.05”. OR, Odds ratio; CI, Confidence interval; CEA, Carcinoembryonic antigen; SII, Systemic immune inflammation index.

### 3.3 Establishment and verification of pCR nomogram model for predicting breast cancer after neoadjuvant therapy

**Figure 1:**
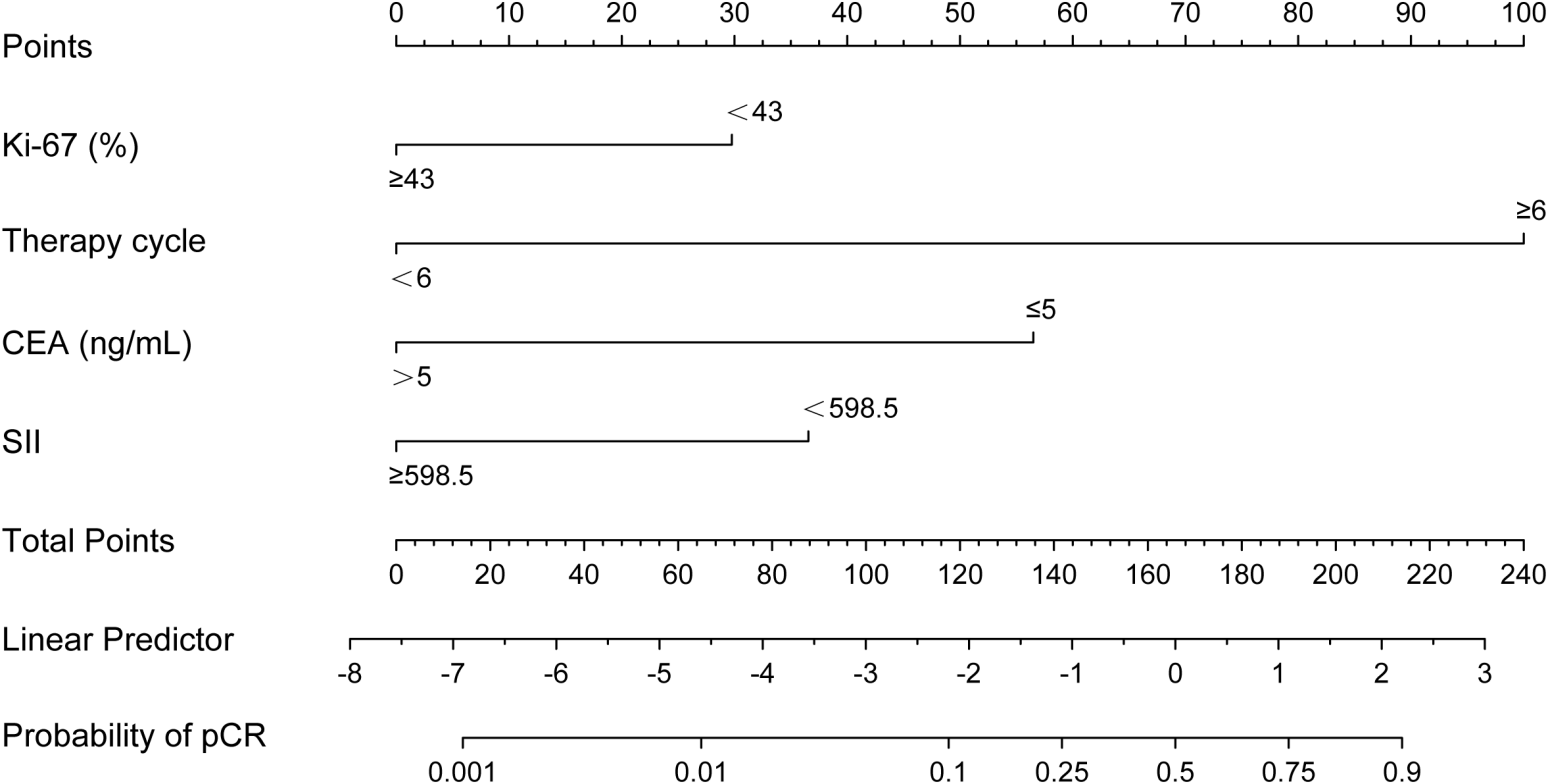
** **A nomogram for predicting pathological complete response after neoadjuvant chemotherapy. CEA, Carcinoembryonic antigen; SII, Systemic immune inflammation index.

**Figure 2:**
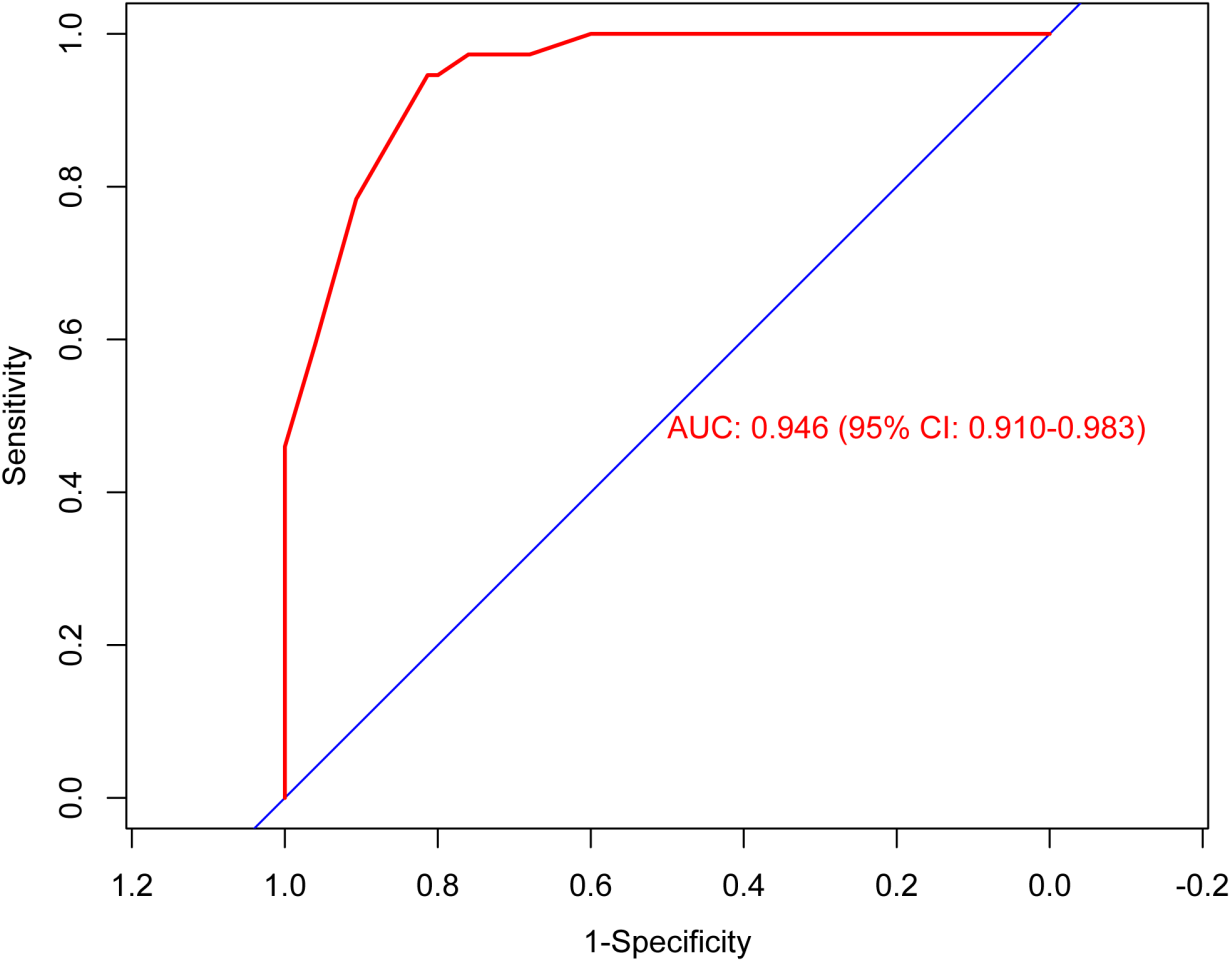
Receiver operating characteristic curve. AUC, Area under the curve; CI, Confidence interval.

**Figure 3:**
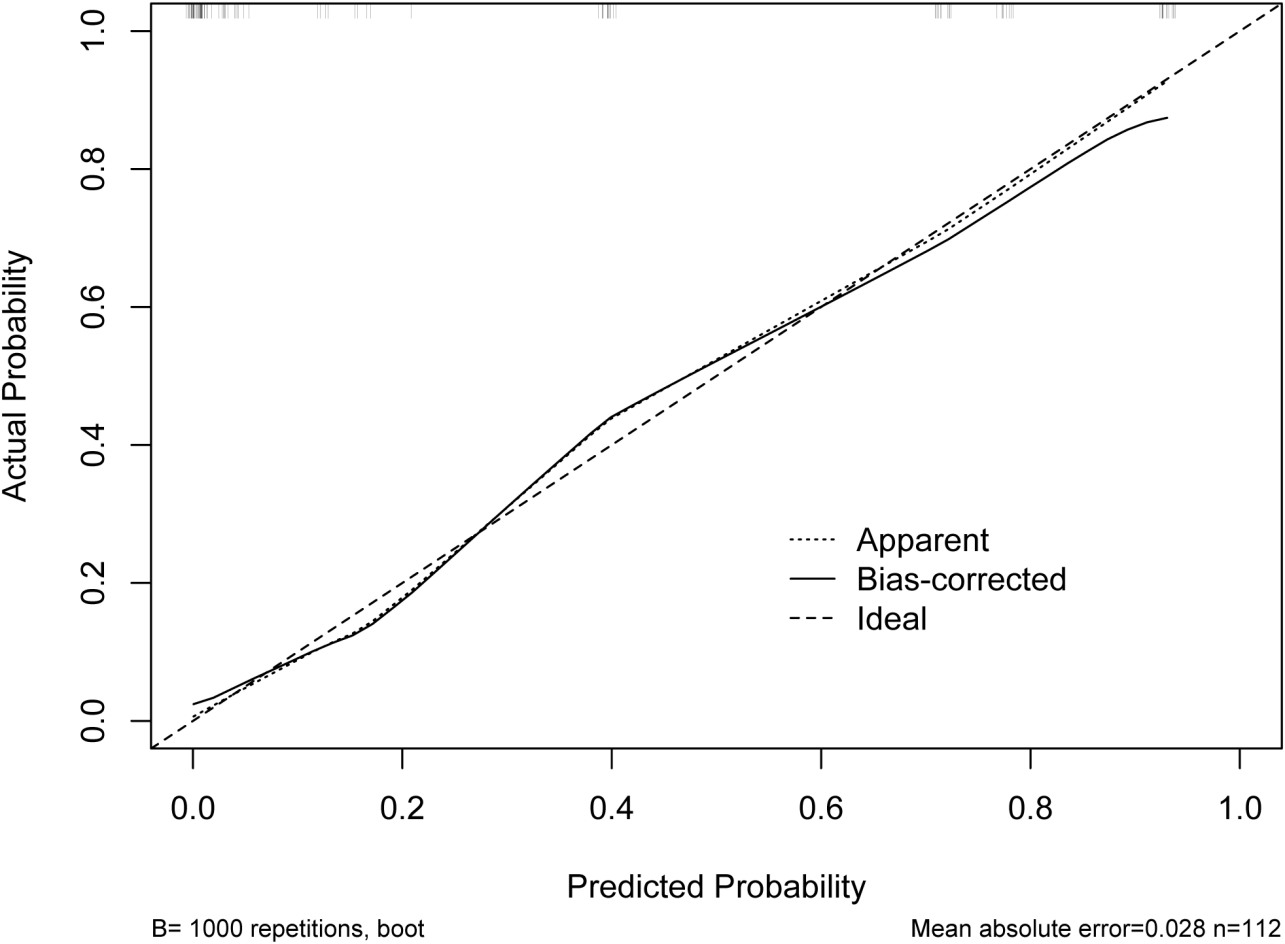
Bootstrap validation curve.

**Figure 4:**
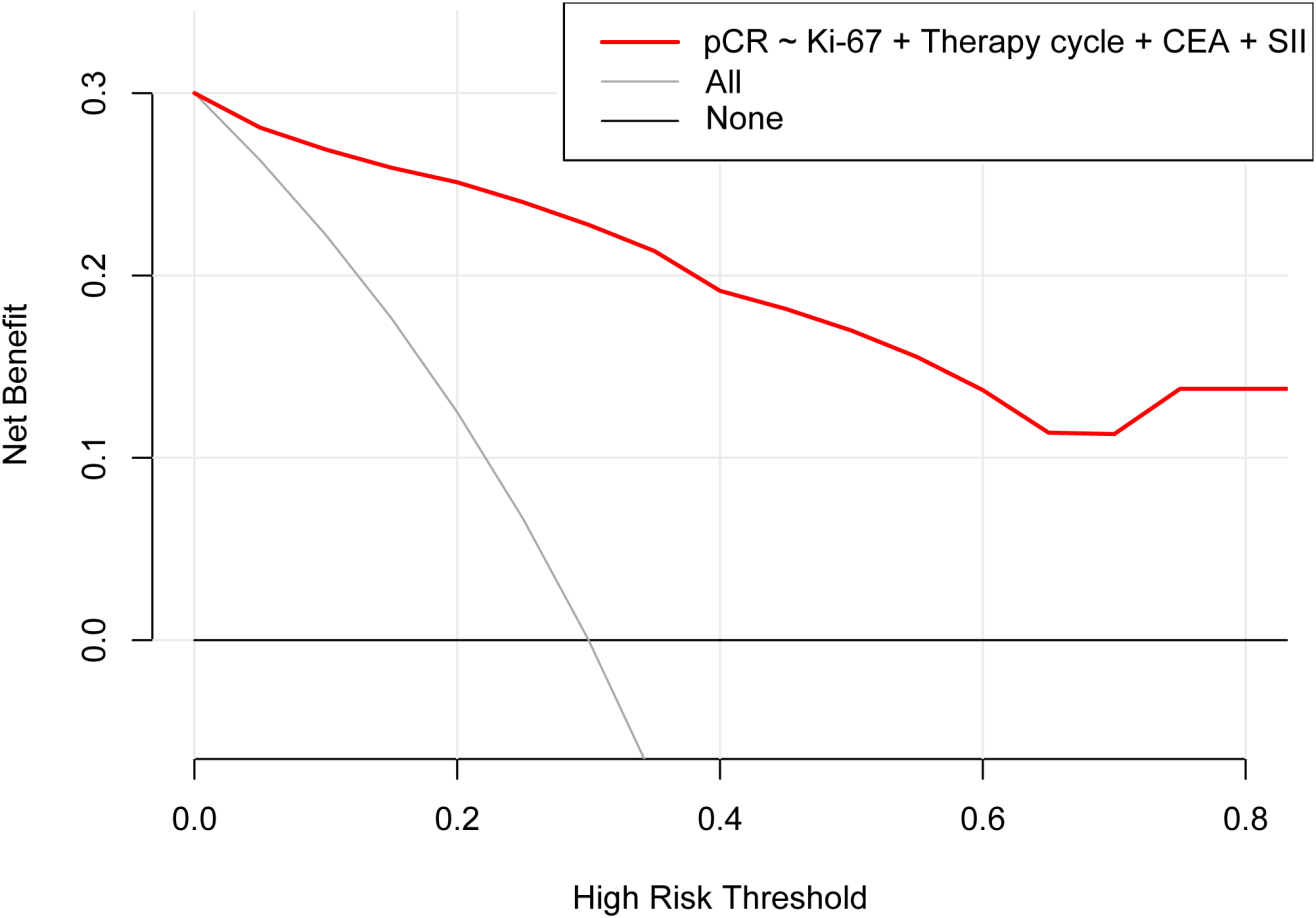
Decision curve analysis. pCR, Pathological complete response; CEA, Carcinoembryonic antigen; SII, Systemic immune inflammation index.

**Figure 5:**
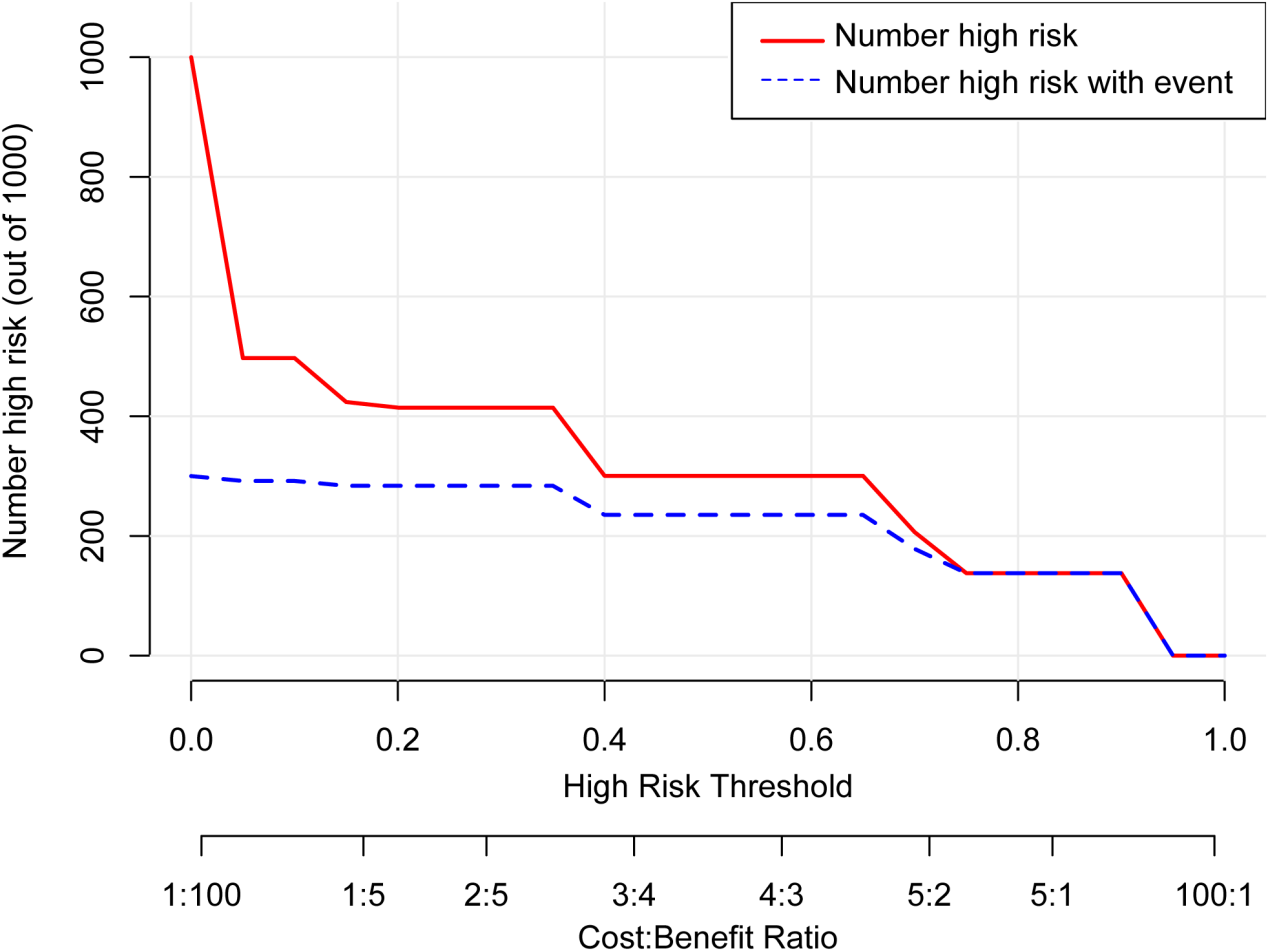
Clinical impact curve.

According to the results of multivariable Logistic regression analysis, a nomogram based on Ki-67, therapy cycle, CEA and SII was established to predict pCR after neoadjuvant therapy for breast cancer (**Figure 1**). ROC curve of the nomogram showed that the area under curve AUC) was 0.946 (95% confidence interval: 0.910-0.983) (**Figure 2**), suggesting that the prediction ability of the model was accurate. We further verified the prediction model by Bootstrap calibration curve, and the results showed that the nomogram had a good discrimination (mean absolute error was 0.028) (**Figure 3**). In addition, we performed DCA curve and CIC curve, and the results showed that the nomogram we constructed had a good ability to predict pCR after neoadjuvant chemotherapy for breast cancer (**Figures 4**, **5**).

## 4 Discussion

This study investigated the role of SII in pCR after neoadjuvant chemotherapy for breast cancer, and analyzed other factors that might affect pCR. Finally, according to the results of Logistic regression analysis, a nomogram prediction model based on SII was established, and the effect of the nomogram on predicting pCR was verified by various internal verification methods.

With the deepening of research, the role of immune and inflammatory responses in tumorigenesis and prognosis evaluation has gradually attracted attention. In the process of tumor invasion, its antigenicity and the release of tissue factors can activate the immune system and play an anti-tumor role through a variety of ways. NLR, PLR, LMR and SII are commonly used new markers and have been proved to be risk factors for poor prognosis of various tumors. However, compared with NLR, PLR and LMR, SII seems to have a greater independent prognostic value and can more fully reflect the balance between immune function and inflammatory response in patients (21, 22). Because SII is a comprehensive indicator of the other three inflammatory indices, high SII may be attributed to changes in these cell counts. Elevated SII indicates an increase in platelets and neutrophils, or a decrease in lymphocytes, indicating that the body’s inflammatory response is enhanced, while immunity is weak. As a hematological parameter, SII is not only easy to obtain, but also easy to detect repeatedly. In clinical practice, SII has been shown to be a prognostic factor for predicting survival outcomes in a variety of cancer patients (10, 23). Previous studies have shown that high SII is closely related to shorter prognosis and higher recurrence rate in breast cancer patients undergoing neoadjuvant chemotherapy (24). Moreover, in patients with HER-2^-^ or triple-negative breast cancer, high SII is positively correlated with shortened survival (25, 26). These results suggest that high SII is a risk factor for poor prognosis in breast cancer patients. The application of neoadjuvant therapy for breast cancer has greatly improved the prognosis of patients, and the achievement of pCR after neoadjuvant therapy is also significantly related to the prolongation of patient survival. In our study, we evaluated the effect of SII on pCR after neoadjuvant therapy in breast cancer patients, and the results showed that high SII was associated with a lower pCR rate. Moreover, Logistic regression analysis also showed that SII was an independent predictor of pCR, which was consistent with previous studies (6, 24–26), indicating that SII had important clinical significance in predicting the efficacy and prognosis of neoadjuvant chemotherapy for breast cancer.

A nomogram is a simple and effective tool for predicting outcomes (27). The nomogram model established based on the results of regression analysis can well predict the pathological response of tumor patients after neoadjuvant therapy (28, 29), and the prediction effect of these models is relatively good. Multivariable regression analysis in this study showed that Ki-67, therapy cycle, CEA and SII were independent factors affecting pCR in patients. Based on the above four indicators, we established a nomogram model that can predict pCR. The AUC value of the model is 0.946, indicating that the nomogram model had good accuracy in predicting pCR. We also verified the nomogram by calibration curve, DCA curve and CIC curve. The results showed that our nomogram model had good prediction ability. Therefore, this study provides a simple and feasible predictive model for pCR in breast cancer patients after neoadjuvant chemotherapy.

However, there are still some limitations in this study. First, this study is a single-center, small-sample retrospective analysis, which may be interfered by confounding factors. Therefore, prospective studies with large samples are still needed to verify the results of this study. Secondly, this study is a retrospective analysis, and it is difficult to obtain more comprehensive clinical data of patients. Therefore, only routine clinicopathological factors and hematological parameters were analyzed. Third, there is no dynamic monitoring and analysis of SII. Finally, due to the lack of external data, the nomogram model established in this study only uses internal verification, and no external verification is performed. Therefore, in the follow-up study, it is necessary to increase the sample size and incorporate more research indicators to improve the prediction model.

## 5 Conclusion

This study clarified the role of SII in pCR after neoadjuvant chemotherapy for breast cancer. Based on Logistic regression analysis, it was found that Ki-67, therapy cycle, CEA and SII were independent factors affecting pCR after neoadjuvant chemotherapy in breast cancer patients. The nomogram model based on Ki-67, therapy cycle, CEA and SII showed a good ability to predict pCR after neoadjuvant therapy.

## Data Availability

The original contributions presented in the study are included in the article/supplementary material. Further inquiries can be directed to the corresponding author.
